# Correction: Freeform imaging systems: Fermat’s principle unlocks “first time right” design

**DOI:** 10.1038/s41377-021-00554-1

**Published:** 2021-05-28

**Authors:** Fabian Duerr, Hugo Thienpont

**Affiliations:** Brussels Photonics, Department of Applied Physics and Photonics (B-PHOT TONA) Vrije Universiteit Brussel, Pleinlaan 2, 1050 Brussels, Belgium

**Keywords:** Other photonics, Applied optics, Optical techniques

Correction to: *Light: Science & Applications* **10**, 95

10.1038/s41377-021-00538-1 published online 06 May 2021

After publication of this article^1^, it is noticed the Author contributions and Funding sections are missing from the back matter. They are hereby provided below.

The original article has been updated.

## References

[1] Duerr F, Thienpont H (2021). Freeform imaging systems: Fermat’s principle unlocks “first time right” design. Light Sci. Appl.

