# Supplementary material for: Freeform imaging systems: Fermat’s principle unlocks “first time right” design
Source: Light Sci Appl. 2021 May 6;10:95. doi: 10.1038/s41377-021-00538-1 (PMC8102611; doi:10.1038/s41377-021-00538-1)
Supplement: Supplementary file 1 — Supplementary Information for Freeform imaging systems: Fermat’s principle unlocks ‘first time right' design [file 41377_2021_538_MOESM1_ESM.pdf]

Supplementary Information for

Freeform imaging systems: Fermat's principle unlocks 'first time right' design

Fabian Duerr† and Hugo Thienpont

Brussels Photonics, Department of Applied Physics and Photonics (B-PHOT TONA)
Vrije Universiteit Brussel, Pleinlaan 2, 1050 Brussels, Belgium

---

Summary: The Supplementary Information provides the background required for hands-on experience with our 'first time right' design method. The user application that we provide, although limited to the design of imaging systems based on three freeform mirrors, clearly illustrates the design approach and its unique strengths. Since the focus of the application is on systems with a moderate field of view (limited to up to 8x8 degrees), there is no explicit user control for field-only-dependent aberration terms (distortion) and the aperture stop position is fixed at the first mirror. The user application has been developed in MATLAB, was partially compiled in C++, and is shared via MATLAB Web App Server through the following permanent link:

http://first-time-right.b-phot.org/webapps/home/session.html?app=FTR

Remark: The application runtime is mainly dominated by the web-based and graphical interfaces, whereas the actual 'first time right' calculations only take a fraction (~0.4s) of the overall time. The actual runtime further depends on the user's internet connection and the server workload.

The data that support the findings of this study are available from the corresponding author upon reasonable request.

The supplementary information in this document is organized as follows:

Note 1 provides a detailed description of the application's Graphical User Interface.

Note 2 summarises how to use the MATLAB web application and explains how a user can reproduce the results shown in Fig. 5 of the paper to become familiarized with the user application.

Note 3 details a brief literature review of freeform three-mirror systems and provides the users with a selection of additional design examples.

Supplementary movie: comprehensive video tutorial in real-time for the web application.

†Corresponding author: Fabian Duerr, fduerr@b-phot.org

Supplementary Note 1: the application's graphical user interface

Figure S1 shows a screenshot of the graphical user interface (GUI) of the 'first time right' MATLAB web application immediately after starting up.

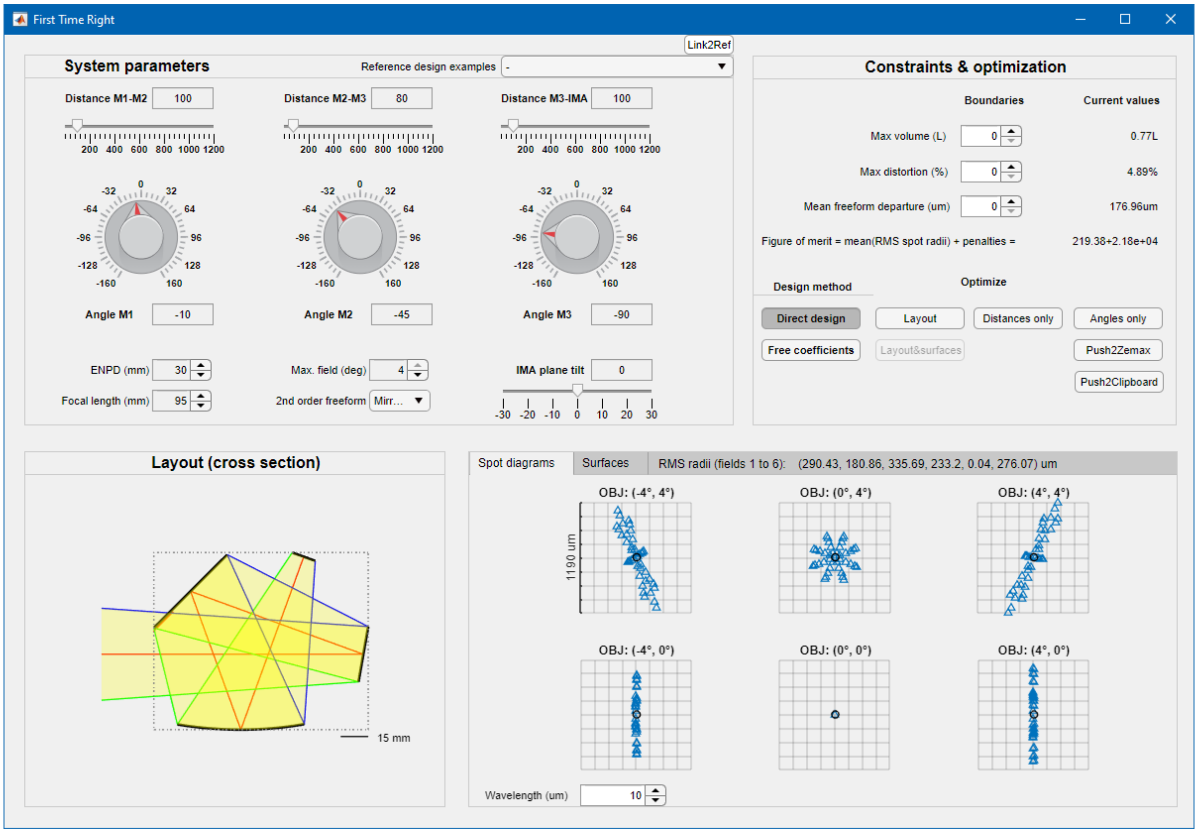

Fig. S1: The GUI of the 'first time right' MATLAB Web App consists of four main windows: ***System parameters*** *(top left, 1.1),* ***Layout*** *(bottom left, 1.2),* ***Evaluation tools*** *(bottom right, 1.3) and* ***Constraints & optimization*** *(top right, 1.4).*

It consists of four main windows: **1.1 System parameters** (Fig. S1, top left), **1.2 Layout** (Fig. S1, bottom left), **1.3 Evaluation tools** (Fig. S1, bottom right) and **1.4 Constraints & optimization** (Fig. S1, top right). All functionalities are explained below.

1.1 GUI of the 'first time right' software application - System parameters

Distances: The distances in mm between mirror 1 (M1), mirror 2 (M2), mirror 3 (M3) and the image plane (IMA) can be changed within the given range from 1 to 1200 mm either through text fields or using the sliders underneath the fields. The values of the text fields and sliders are linked.

Angles: The individual alignment (tilt angle about the y-axis in degrees) of each of the mirrors M1 to M3 can be varied within the given range of -160 to 160 degrees either by using the knobs or through the text fields underneath. The values of the text fields and knobs are coupled. Counterclockwise rotations are defined as positive and clockwise rotations as negative angles. Special care should be taken with respect to the relative mirror orientations. For example, a rotation angle of -70 or 110 degrees (mirror angle flipped by 180 degrees) would not change the system in

case of a flat mirror would be flipped. However, in cases where curved surfaces are flipped, it is therefore important to only apply tilt angles that ensure that the rays intersect with the same sides of the mirrors as if all tilt angles were zero.

Specifications: There are three text fields that allow the user to specify the entrance pupil diameter in mm, the focal length in mm, and the maximum field angle α (here limited to four degrees) for a squared field of view in the x- and y-directions and thus a diagonal full field of view of $2\alpha\sqrt{2}$. The *2nd order freeform* drop-down menu allows to select for which of the three mirrors individual curvatures in x- and y-direction will be calculated. The other two (not selected) mirrors have equal 2nd order coefficients (thus a base curvature) in x- and y-direction, respectively. All surfaces are provided as XY-polynomial coefficients as direct consequence of the design method.

IMA plane tilt: The relative image plane (IMA) tilt angle in degrees can be varied either through the text field or by using the slider underneath. A tilt angle of zero degrees means that the image plane is oriented such that its normal vector coincides with the on-axis chief ray. The value of the text field and that of the slider are coupled.

1.2 Application GUI - Layout

The layout window in Fig. S1. shows the cross-section in the symmetry x-z-plane. The semi-transparent yellow regions indicate the overall extend of the light rays' bundle for all fields combined from object to image. The analytically traced rays are the central chief ray (in red), and the confining boundary rays (in green and blue). They always start from left and proceed to right in horizontal direction. The mirrors and the image plane are plotted as black lines. Their maximum diameter is estimated by the ray mapping functions for all fields. The rectangle in black dotted lines indicates the cross-section of the area surrounding the mirrors. It is used to calculate the volume of the optical system as the product of the surface area of this box times the maximum diameter of the mirrors M1 to M3. In case any of the surfaces (mirrors or image surface) blocks the overall ray bundle, this (partial) overlap is visualized in red. In the start-up configuration (see Fig. S1), the second mirror is partially blocking the light rays travelling towards the first mirror. We use a MATLAB built-in tool that is based on the calculation of pairwise intersections of polyshape objects to automatically detect obscuration and to verify if any mirror or image plane causes obstruction along the light path. Here, the known ray mapping functions for each mirror can be used for such calculations as the "analytic ray tracing" provides all the necessary information. Alternative obscuration detection and elimination methods can of course be implemented following reference[1].

1.3 Application GUI - Evaluation tools

The evaluation tools window in Fig. S1 offers two tabs. One allows to visualize the optical performance, the other the surfaces of the optical system. The first tab *Spot diagrams* (default) shows the ray intersects (spots) with the image plane for six predefined fields, which scale with the user defined maximum field of view angle. The calculated RMS radii of the fields 1 to 6 (in

micrometres) are listed next to the tabs at the top. At the bottom of the tab is a spinner text field that allows to define a wavelength that updates the corresponding Airy disk in the respective spot diagrams. The second tab *Surfaces* provides the surface sag (top row) in millimetre, and the freeform departure (bottom row) in micrometre for each of the mirrors M1 to M3.

1.4 Application GUI - Constraints and optimization

Constraints: There are three text fields where the user can specify boundary constraints. First, the maximum allowed optical system volume in litres; second, the maximum allowed distortion in percent; and third, the maximum allowed mean freeform departure in micrometres. Next to these three text fields, the current values for the volume, distortion and mean freeform departure are displayed. Setting one of these boundary values to zero means that this boundary value is unconstrained. If the current design exceeds any of the set boundary values, that value is highlighted in red and imposes a penalty term in the merit function.

Merit function: The merit function value is the mean RMS spot size of the six fields displayed in the spot diagrams (see 1.3 Evaluation tools). It is a performance indicator plus penalty terms in case any of the boundary constraints has been breached or if obscuration appears in the design.

Design method and optimization: There are two possible options under design method, that is *Direct design* or *Free coefficients*. In *Direct design* mode, any changes to the system parameters will trigger immediate recalculation of the system following the steps outlined in the paper. There are three possible buttons and thus variable sets to optimize the reduced direct design parameter space: *Layout*, *Distances only* and *Angles only*. *Layout* means that all six (seven if the IMA tilt value is not zero) distances and angles are varied during the optimization. *Distances* or *Angles only* mean that either only the three distances or the three angles (four if IMA tilt is not zero) are varied during the optimization process. In all three cases, the figure of merit is the mean RMS spot radius plus penalty terms if any given boundary condition has been breached. Once started, the iterations of the optimization and the current figure of merit will be shown at the bottom of the *Constraints & optimization* window. The stop button can be clicked to interrupt the ongoing optimization.

In *Free coefficients* mode, all surface coefficients (surfaces shapes) are frozen and thus it is not recommended to change the system parameters (distances and tilts) at this stage. The *Layout&surfaces* button triggers an optimization where all six (or seven if the IMA tilt value is not zero) system parameters and the 40 non-zero surfaces coefficients are varied. Here, the figure of merit is the squared sum of all ray deviations from the spot centre in x- and y-direction for each respective field. The *Current figure of merit* value, however, is still the mean RMS spot radius in order to better illustrate the optimization progress. It is important to note that the first 50 function executions are taking place in a background process before the optimization progress will show a first *Current figure of merit* value. The stop button can be clicked at any time to interrupt the ongoing optimization process. This will happen as soon as the next iteration is finished. Once the optimization finishes, the GUI is automatically updated.

Finally, the button *Export system* allows the user to save all relevant system data including the distances, tilt angles and surface coefficients to a text file. Even though data transfer to optical ray-tracing software such as Zemax is currently not possible through our web application, the text export approach will allow users to manually import the system into a commercial ray-tracing software or through a user script. All surface coefficients are provided as XY-polynomial coefficients as a direct consequence of the presented design method. It is important to emphasise that adding a spherical radius, a conic constant or performing a conversion to alternative surface representations such as Zernike, Forbes or Chebyshev polynomials can be immediately done[2].

Supplementary Note 2: how to use the MATLAB web application

In this note, we explain how a user can reproduce the results shown in Fig. 5 of the paper to become familiarized with the user application. In a first step, the user should select the predefined example *1. Current article (Fig. 5)* in the drop-down menu *Reference design examples* placed next to the *System parameters* header. The layout on display has already been optimized using the *Layout* (*Direct design* state) from a manually adjusted unobscured system that fitted the 60 litres volume constraint and corresponds to the shown system in Figs. 5a and b. A further *Layout* optimization would therefore hardly improve the figure of merit, i.e. the mean RMS spot size. Next, the user should switch the *Design method* mode to *Free coefficients* and then click *Layout&surfaces* in order to perform a final optimization of the directly calculated system. The optimization will start and will show after a delay of approximately 40-60 seconds the progress. After 10 iterations, this optimization will automatically stop and the updated system in the GUI will show the result of Fig. 5c and 5d. The optimization process can be continued and will stop when the maximum number of iterations has again been reached.

An important design consideration is the selection of the system's layout parameters, namely the relative distances and tilt angles. This has been touched upon briefly in the paper. In principle, a user can freely change these values either by editing the numeric fields or by adapting the sliders and knobs. The selection of the *2nd order freeform* term (the mirror that has not a base sphere but different second order coefficients in x- and y-direction) is a further option to consider at an early design stage. This manual option could be easily automated in the future. In cases where the selection of the system parameters does not result in a "suitable" solution, the *Layout* and *Evaluation* will still be updated. Here, "suitable" typically means that the first order nonlinear system of equations has a solution with moderate curvatures on the three mirrors. In such cases, the user should continue changing the system parameters until a suitable solution is found. Even though an immediate *Layout* optimization is always possible in principle, it will not guarantee to find "suitable" solutions directly. In principle, global optimization tools such as global or multiple starting point search, pattern search, genetic algorithm or particle swarm algorithms (for example, see https://www.mathworks.com/products/global-optimization.html) could be used to effectively evaluate the largely reduced parameter space. Typically, a good start for the user is to manually adjust the layout to move closer towards an unobscured well-working system. If there are additional boundary constraints in place, it is certainly helpful to already approach, or ideally drop

below such constraint values. A partial *Distances* or *Angles only* optimization can further help to guide the design process at an early stage where it is evident that either the distances or tilt angles are the main cause for residual obscuration or infringed constraints. Once an unobscured system that (almost) meets the given boundary constraints has been established, a *Layout* optimization helps to further improve the considered geometry. Alternatively, the unobscured system can be readily transformed into an alternative alignment of the mirrors by changing the tilt angles, and later adjusting the distances. Repeating this process in real-time provides a very fast evaluation tool of possible mirror folding layouts with their typical performance potential, volume, distortion and freeform departures.

Supplementary Note 3: additional design examples from literature

We performed a brief literature review of three freeform mirror systems to provide the users with a preselection of additional design examples. The references from literature that we have covered and discussed in this Supplementary Information Note are only a selection of examples with a clear emphasis on general freeform design methods and strategies. They do not represent a complete literature survey. We do not intend to rate or value other design methods through the presented comparisons. The idea of using various examples from literature was to demonstrate the effectivity and versatility of our approach. The references only provided the overall system specifications and the rough layout of how the mirrors are placed in space with respect to each other. We did not estimate or measure any distances nor angles when redesigning the systems. The purpose of the web application that we share is to highlight the novel design method as an excellent initial system calculator. It was not our intention in this Supplementary Information to focus on explicit applications or on their specific constraints. The user can easily access these examples through the drop-down menu *Reference design examples* located next to the *System parameters* header. The click on one of these examples will automatically update all relevant parameters and constraints in the user application. These parameters have been manually adjusted to create an unobscured system that matches the reference's layout and fulfils the given constraints, if any. Also, a *Layout* optimization of 100 iterations has been already performed to further improve the considered geometrical arrangement of the mirrors, which is thus the final state shown in the web application. The solutions shown are probably not the best that can be achieved, but once the performance matched or exceeded the reported results we have stopped the design process. Finally, the button *Link2Ref* just above the drop-down menu will take the reader to the website of the respective reference paper for further reading. The following design examples are made available:

3.1 Design example current paper (Fig. 5)

The first example[2] *1. Current paper (Fig. 5)* has been discussed in the main article and corresponds to the results shown in Figs. 5a and b, and Figs. 5c and d after further optimization using the *Layout* optimization routine (see Supplementary Note 2: how to use the MATLAB web application). An interesting further result for a very similar system with the same specifications, except for the volume constraint, has been designed by Menke using particle swarm optimization[3].

3.2 Design examples in Light: Science & Applications 6, e17081 (2017)[4]

In this paper, the authors present a framework based on a point-by-point design process that can automatically obtain high-performance freeform systems and requires only a combination of input planes for the mirrors. The first example of the article works under the long-wave-infrared spectral band and has a 53 mm entrance pupil diameter, an f-number of 1.798 and covers an 8×8 degrees field of view. In the original system, the secondary mirror (M2) was the aperture stop. Their final design result is shown in Fig. S2.

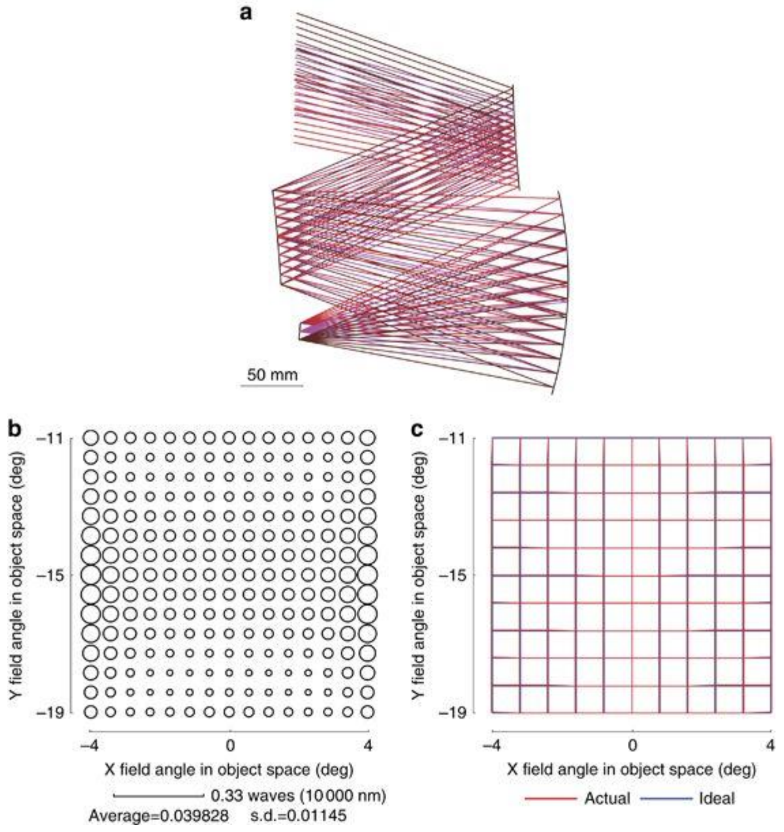

Fig. S2: Original Fig. 6 in reference[4] with caption: "(a) Layout of the final design result. (b) RMS wavefront error of the final design result. (c) Distortion grid of the final design result."

The article does not mention any volume constraints; thus, we estimated the system's volume at about five litres using manual measures, the distortion is limited to about 0.75%.

Our redesign of reference example *2a. doi.org/10.1038/lsa.2017.81 (Fig. 6)* shows the directly calculated solution of a related system with about 3.5 litres volume, well-constrained distortion and a mean RMS spot radius of less than 30 microns, shown in Fig. S3.

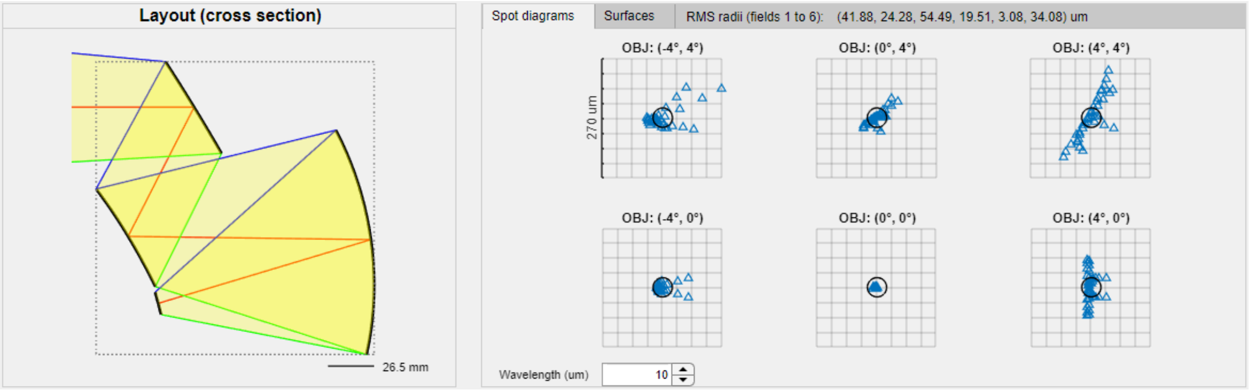

Fig. S3: Reproduced result based on direct calculations for the first given example in reference[4] (original Fig. 6)

Even though the primary mirror is the aperture stop here, this generated starting form can be exported to a ray tracing software where the stop can then be changed to the second mirror. Further optimization quickly results in a final system that matches the given average 0.04 waves RMS wave front error and probably exceeds the reported performance.

The second example in the article is inspired by a similar system that was first designed by Fuerschbach et al[5]. The system works under the long-wave-infrared spectral band with a 30 mm entrance pupil diameter, has an f-number of 1.9 and covers an 8×6 degrees field of view. The second mirror serves as the aperture stop in the original system. Their final design result is shown in Fig. S4. The article does not mention any volume constraints; thus, we estimated the system's volume at about 2.5 litres using manual measures, the distortion is limited to 1%.

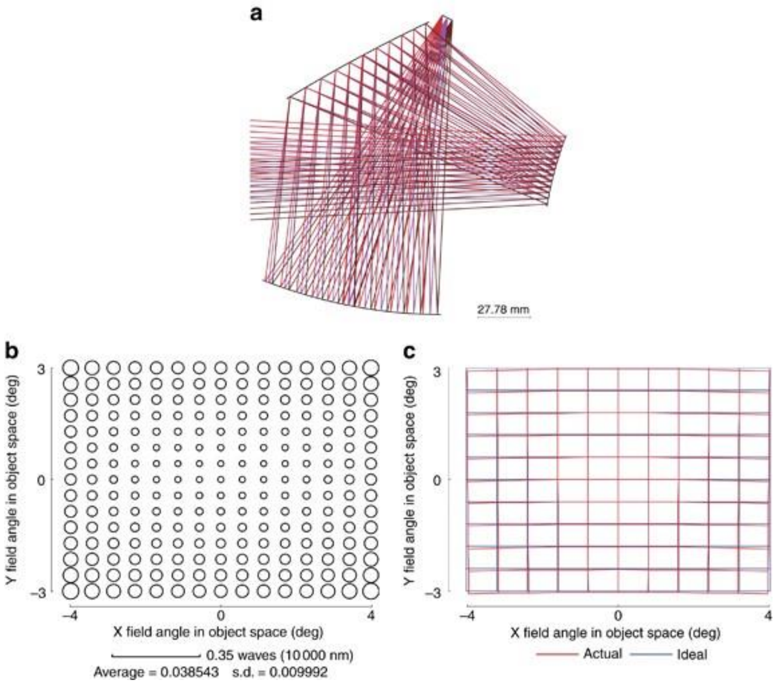

Fig. S4: Original Fig. 7 in reference[4] with caption: "(a) Layout of the final design result. (b) RMS wavefront error of the final design result. (c) Distortion grid of the final design result."

Our redesign of reference example *2b. doi.org/10.1038/lsa.2017.81 (Fig. 7)* shows the directly calculated solution of a related system with a volume of about 2.4 litre, well-constrained distortion and covering a similar but squared 7×7 degrees field of view. In our design we have kept the stop at the primary mirror as it is a good choice for the given alignment. The obtained mean RMS spot radius is about 8.5 microns, which is shown in Fig. S5.

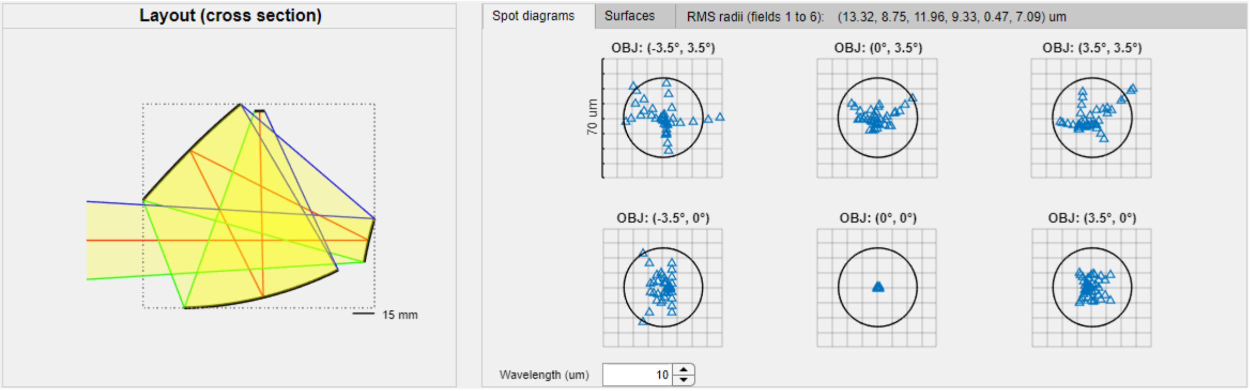

Fig. S5: Reproduced result based on direct calculations for the second given example in reference[4] (original Fig. 7)

The user can now change to *Free coefficients* mode and click *Layout&surfaces* optimization, which results in the system shown in Fig. S6 after 10 iterations taking few minutes.

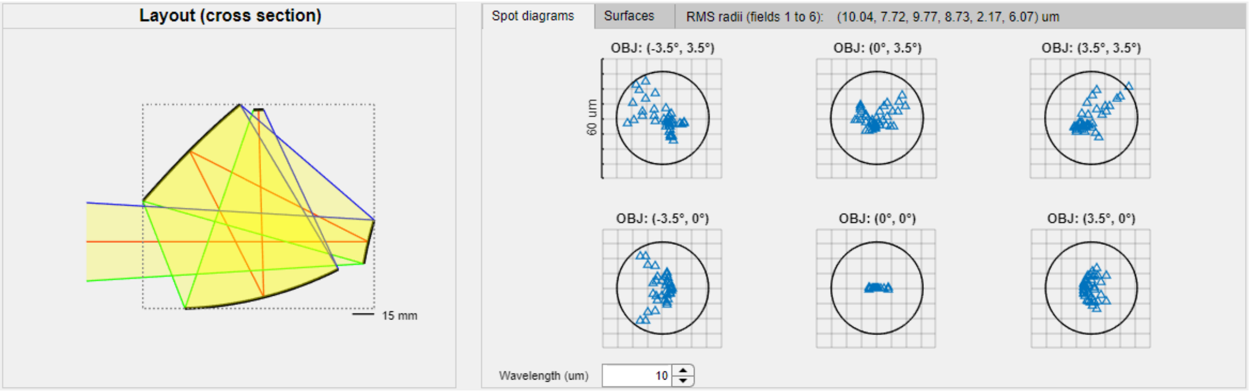

Fig. S6: Result after a final optimization reproducing the second given example in reference[6] (original Fig. 7)

The optimization converges to a final system with a mean RMS spot radius of about 7.4 microns. Once imported into a ray tracing software for evaluation with an 8×6 degrees field of view, the mean RMS wave front error is about 0.032 waves, slightly better than the reported performance.

3.3 Design example in Opt. Express 25, 14598-14610 (2017)[6]

In this article, the authors use a curved image detector in a three-mirror freeform design and evaluate possible performance benefits. Their system has a 100 mm entrance pupil diameter, an f-number of 2.5 and covers a 7.2×7.2 degrees field of view. The volume is constraint to about 50 liters and the maximum distortion is about to 1.33%. For two different freeform polynomial functions the best results were achieved with a toroidal detector, shown in their Table 3. (Fig. 7)

Table 3. Image quality indicators

Parameter	Legendre		Square Zernike	
	min	max	min	max
RMS spot radius, μm	1.4	5.9	5.8	9.1
Circumcircle radius, μm	2.3	10.9	8.6	15.9
Energy concentration in 5μm radius, %	85.3	93.8	27.2	88.7

Fig. S7: Original Table 3 in reference[6] with caption: "Image quality indicators"

Here, we try to obtain similar results however with a flat image detector. Our redesign of reference example *3. doi.org/10.1364/OE.25.014598 (Fig. 4)* shows the directly calculated solution of a similar system with both volume and distortion well-constrained. The obtained mean RMS spot radius is about 10.1 microns, which is shown in Fig. S8.

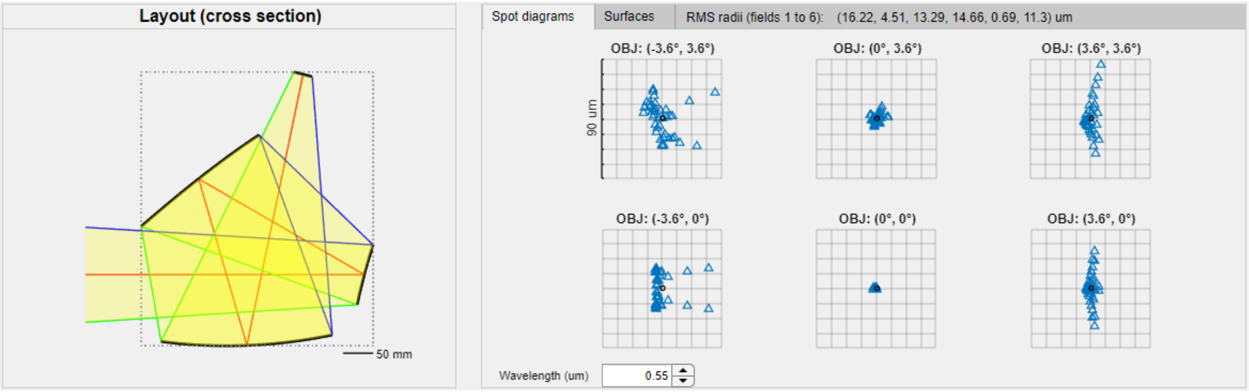

Fig. S8: Reproduced result based on direct calculations for the example in reference[6] (original Figs. 3 and 4)

The user can now change to *Free coefficients* mode and click *Layout&surfaces* optimization, which results in a further improved system that is shown in Fig. S9.

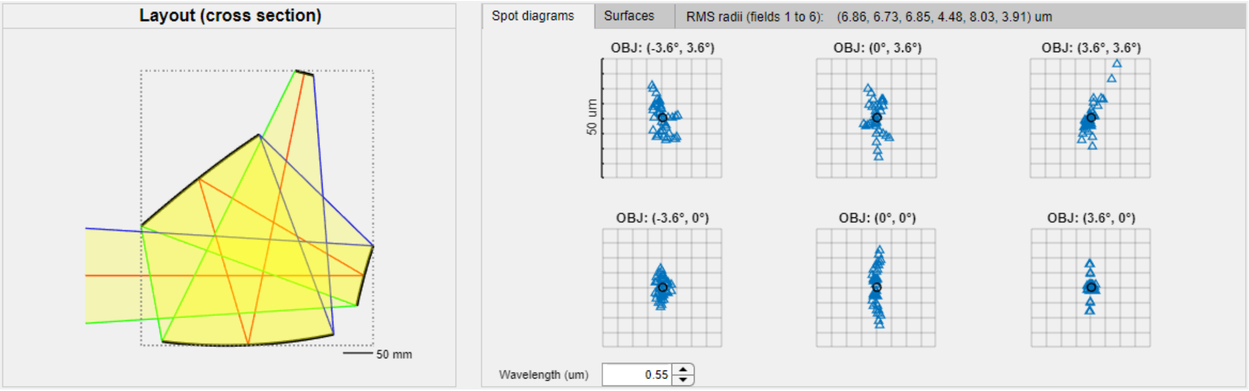

Fig. S9: Result after a final optimization reproducing the example in reference[6] (original Figs. 3 and 4, Table 3)

The resulting RMS spot radii range from 3.9 to 8 microns (3.4 to 7.9 microns based on real ray tracing) for a flat image and thus slightly exceeding the reported Zernike-based results with a toroid image. The maximum RMS spot radius is close to the reported Legendre-based results. If we would allow the image plane to become a spherical surface, the performance will match or eventually

exceed the best reported result for Legendre-based freeform surfaces with a toroidal image. A closely related discussion on the choice of surface form descriptions and spherical vs. toroidal shaped image detectors is provided by Dave Shafer in one of his slideshows[7].

3.4 Design example in Opt. Express 25, 10016-10030 (2017)[8]

The second example from this article targets a compact folding structure three mirror system working in the mid and long wave infrared (MWIR and LWIR) spectrum and has a 200 mm entrance pupil diameter, a f-number of 1.55 and covers a 1.774×1.331 degrees field of view. The system specifications have been obtained from a proceedings paper by H. Zhu[9]. Here, the stop is placed before the first mirror. As the article focuses on an initial design method for freeform systems based on Gaussian brackets and Nodal aberration theory, the intermediate system shown in Fig. S10 (original Fig. 6) features well-balanced spot sizes, which can serve as a good starting point for further optimization.

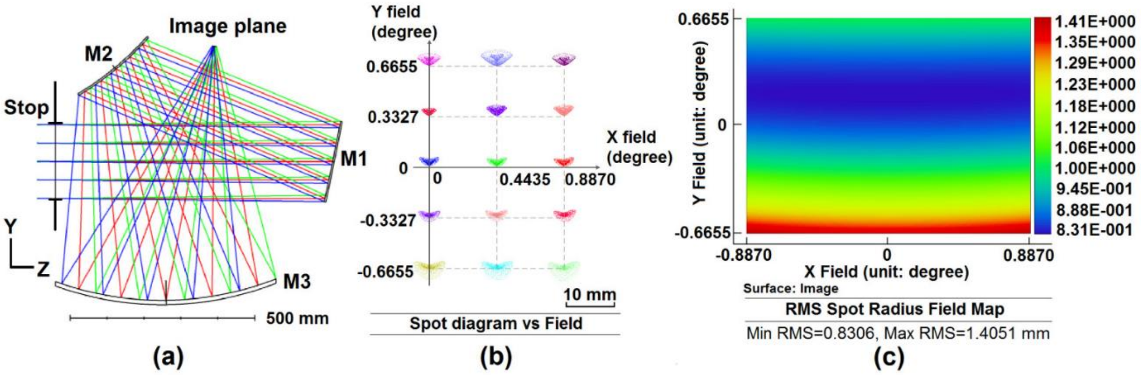

Fig. S10: Original Fig. 6 in reference[8] with caption: "System performance of the compact folding structure TMA system (a) System layout; (b) Spot diagram with field; (c) RMS Spot radius map with field."

An optimized design [9] of the same system is shown in Fig. S11 that achieves close to diffraction-limited performance in both the MWIR and LWIR band.

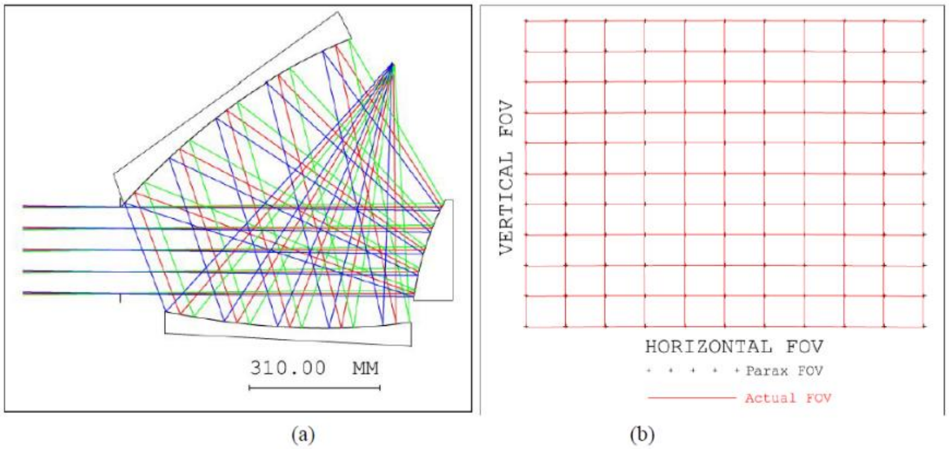

Fig. S11: Original Fig. 2 in reference[9] with caption: "(a)Optical system layout and (b) Grid distortion"

Both articles do not mention any volume constraint; however, we estimated the system's volume at about 80 liters and limited the distortion to 0.5%. Our redesign of reference example *4. doi.org/10.1364/OE.25.010016 (Fig. 6)* shows the directly calculated solution of a similar system but where the stop is placed at the first mirror (the stop could be moved in front) with well-constrained volume and distortion and covering a larger squared 2×2 degrees full field of view. The obtained mean RMS spot radius is about 7.9 microns, which is shown in Fig. S12.

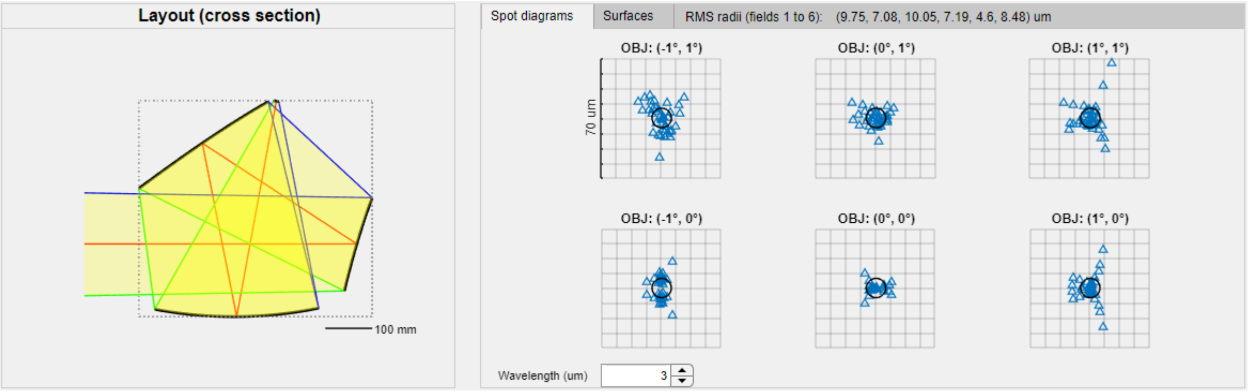

Fig. S12: Reproduced result based on direct calculations for the design example discussed in references[8,9]

The user can now change to *Free coefficients* mode and click *Layout&surfaces* optimization, which results in a further improved system that is shown in Fig. S13.

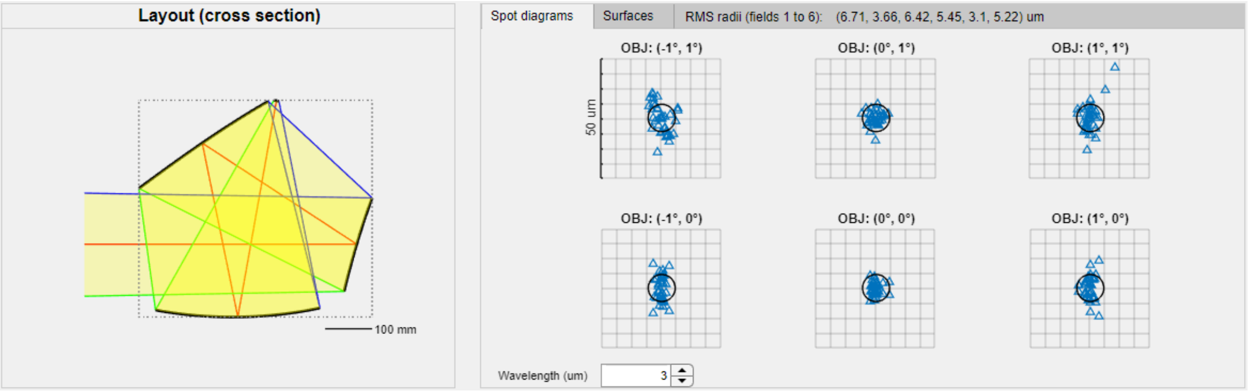

Fig. S13: Result after a final optimization reproducing the example discussed in references[8,9]

The optimization yields a final system with a mean RMS spot radius of about 5.1 microns. Once imported into a ray tracing software, the evaluation confirms a close to diffraction-limited performance in the MWIR band.

3.5 Design examples in Opt. Express 27, 15251-15261 (2019)[10]

This article provides three examples that all share a 100 mm entrance pupil diameter, an f-number of 4.5, and that cover a 4×4 degrees field of view. Here, the stop is placed at the second mirror, in contrast to our case where it is positioned at the first mirror. As there are no further boundary limitations discussed in the article, we have estimated the volume constraint at about 15 liters and

set the maximum distortion to 1% for all three cases. As the evaluation is purely geometrical here, we have set the wavelength arbitrarily to 1 micron. Their first design example is shown in Fig. S14. The reported maximum and average RMS spot radii are 145 um and 81 um, respectively.

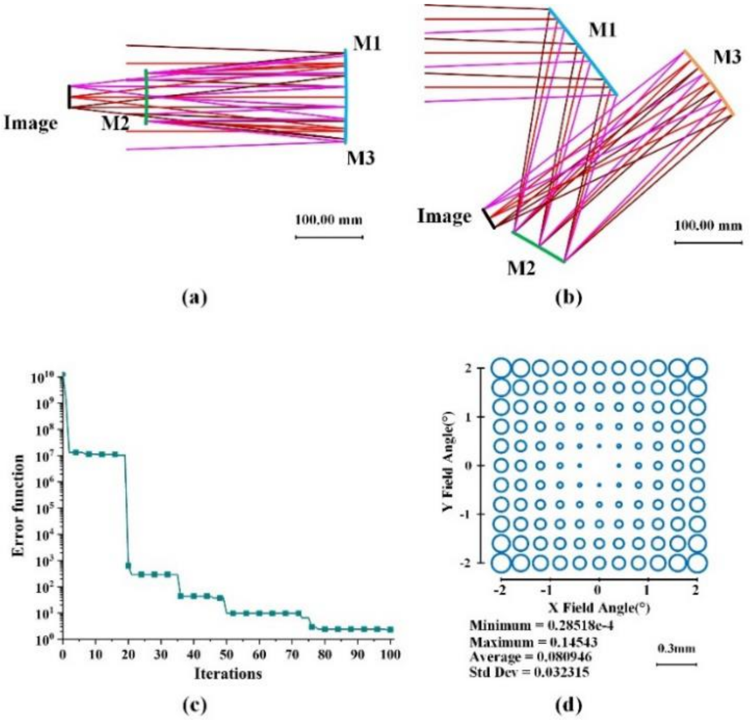

Fig. S14: Original Fig. 3 in reference[10] with caption: "The process of initial system searching for a specified OPC. (a) The initial point, (b) the searching result with V = [-1, −1, 0, 0, 1], (c) the error function with the logarithmic scaling during the searching process, and (d) the RMS spot sizes of the result."

Our redesign of reference example *5a.* *doi.org/10.1364/OE.27.015251* *(Fig. 3)* shows the directly calculated solution of a similar system where the stop is placed at the first mirror with well-constrained volume and distortion. The achieved maximum and average RMS spot radii are about 22 um and 14 um, respectively, which is shown in Fig. S15.

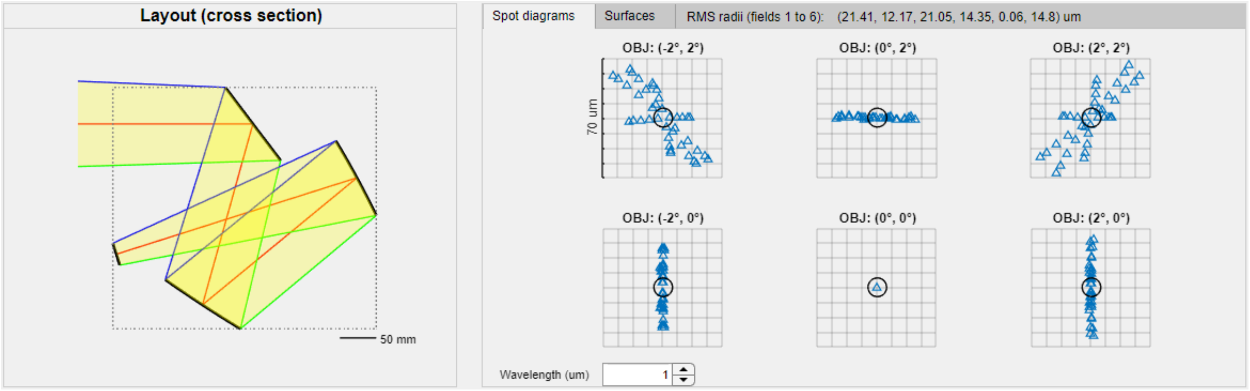

Fig. S15: Reproduced result based on direct calculations for the first example discussed in reference[10]

Their second example targets a system design that should have M2 and M3 close to each other to be fabricated on the same substrate, leaving only a small gap between these two mirrors. Their resulting system is shown in Fig. S16.

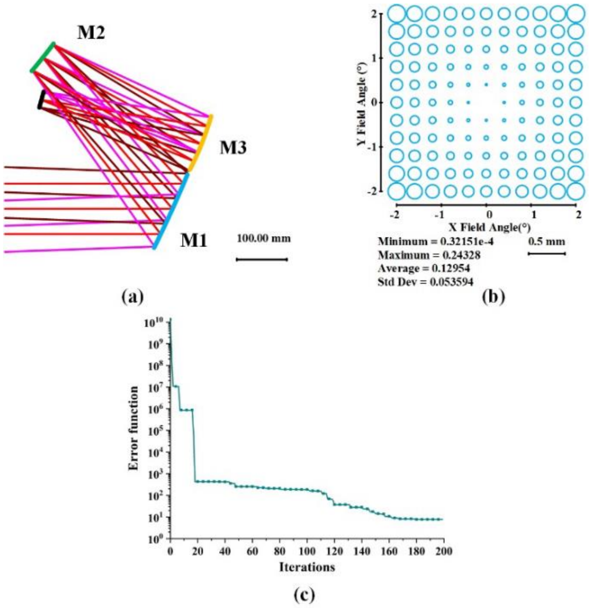

Fig. S16: Original Fig. 4 in reference[10] *with caption: "The result of the automatic OPC optimization is shown in (a), and the RMS spot sizes of the system is shown in (b). It can be seen from (a) that M1 and M3 are close enough to be fabricated on the same material. The error function during the optimization process is in (c), where a block symbol is plotted every 4 iterations and the logarithmic scaling is adopted for the error function"*

The reported maximum and average RMS spot radii are 243 um and 130 um, respectively. We adjusted the position of the two mirrors manually to bring them close together, whereas further conditions on a smooth continuation between the two mirrors could be imposed.

Our redesign of reference example *5b.* doi.org/10.1364/OE.27.015251 *(Fig. 4)* shows the directly calculated solution of a similar system. The achieved maximum and average RMS spot radii are about 25 um and 15.5 um, respectively, which is shown in Fig. S17.

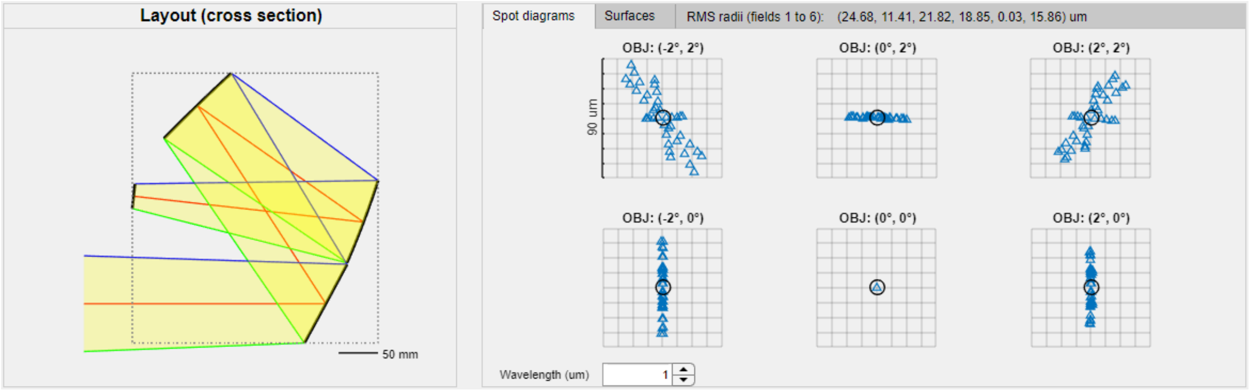

Fig. S17: Reproduced result based on direct calculations for the second example discussed in reference[10]

Their third design example is used to demonstrate the transition from one system geometry to another geometry through an "unfeasible area with obscuration", see Fig. S18b. The reported maximum and average RMS spot radii are 135 um and 81 um, respectively.

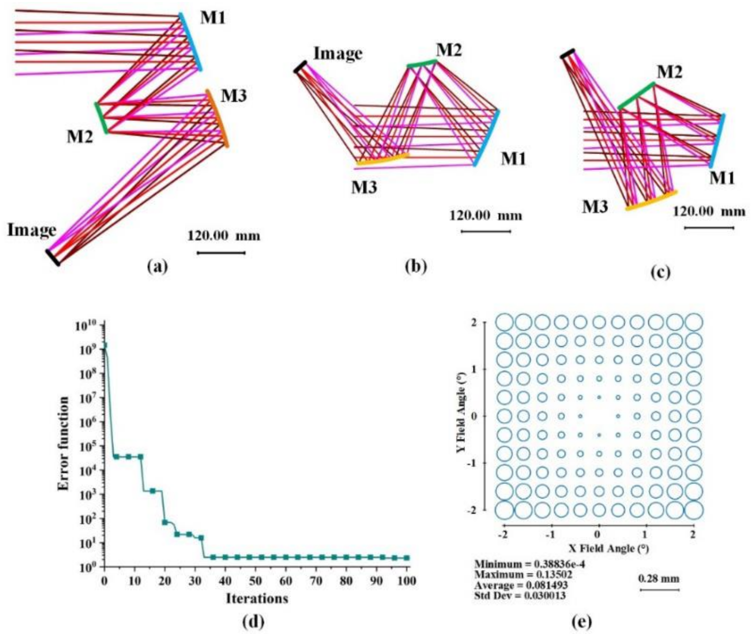

Fig. S18: Original Fig. 5 in reference[10] with caption: "The OPC variation process of Example 3. (a) The starting point, (b) the obscured system in the process, (c) the unobscured result that meets the target, (d) the logarithmic-scaled error function during the automatic optimization, and (e) the RMS spot sizes of the result."

With our user application, we can immediately alter and adjust the mirror alignments that allows to reproduce a system configuration corresponding to sub-figure (c) in Fig. S18.

Our redesign of reference example *5c. doi.org/10.1364/OE.27.015251 (Fig. 5)* shows the directly calculated solution of such a similar system. The achieved maximum and average RMS spot radii are about 22 um and 15 um, respectively, which is shown in Fig. S19.

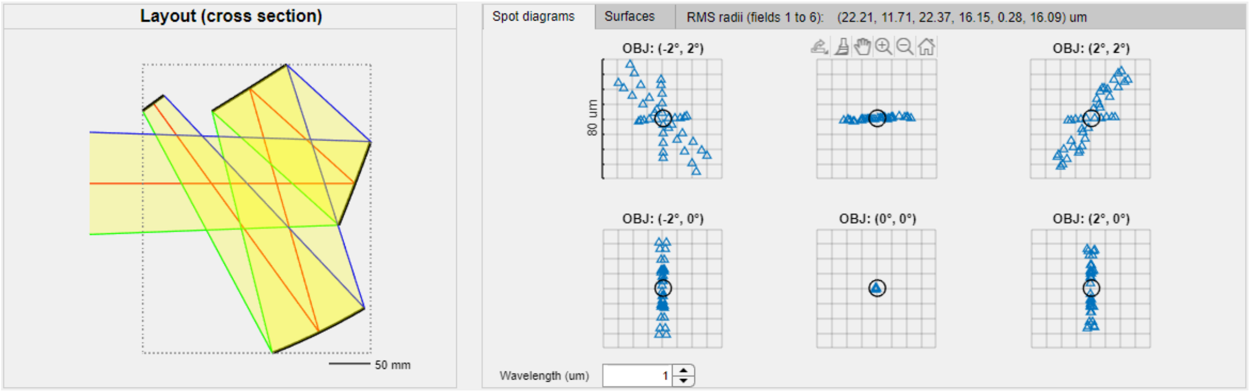

Fig. S19: Reproduced result based on direct calculations for the third example discussed in reference[10]

By comparison, the achieved maximum and average spot sizes are substantially smaller than the reported values in all three directly calculated cases. Clearly, a further improvement could be achieved when using these ‘first time right’ starting points for subsequent optimization.

3.6 Design examples in Proc. SPIE 10690, 106901D (2018)[11]

This article provides a survey of the three-mirror freeform imager solution space and presents various design examples that are calculated based on a method that combines several concepts and techniques from literature to analytically generate unobscured freeform starting point designs that are corrected through third-order image degrading aberrations.

The first considered example from a “Smaller etendue survey run” has a focal length of 100 mm, an aperture of F/5 and covers a full field of view of 2×2 degree with the stop placed at the first mirror. We have estimated the volume constraint at about 0.12 liters and the maximum distortion is limited to 1%. As the evaluation is purely geometrical here, we have set the wavelength arbitrarily to 0.5 micron. Their first example (original Fig. 9) is shown in Fig. S20.

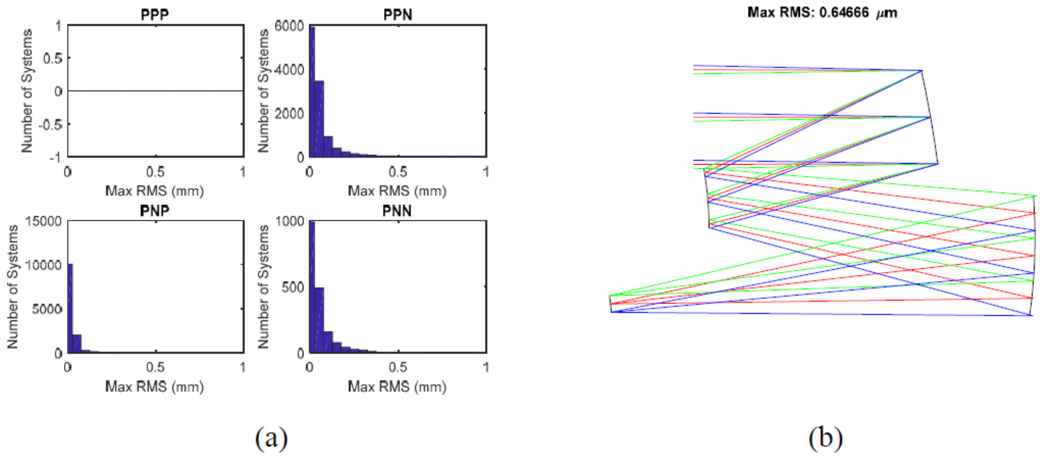

Fig. S20: Original Fig. 9 in reference[11] with caption: “(a) Four performance histograms representing the four distinct tilt angle sign combinations for the survey run with an effective focal length of -100 mm, flat field solution 1, F/5, FFOV 2 degrees by 2 degrees. The systems are binned according to the worst field point RMS spot size. (b) An optical layout of the best performing, analytically designed system from this survey run, along with its max RMS spot size of 0.65 microns.”

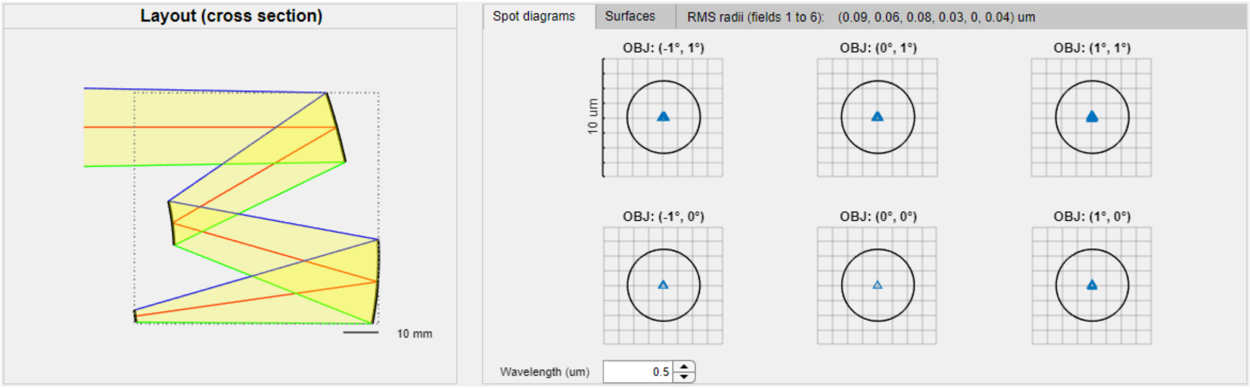

Fig. S21: Reproduced result based on direct calculations for the example of the original Fig. 9 discussed in reference[11]

Our redesign of reference example *6a. doi.org/10.1117/12.2314403 (Fig. 9)* shows the directly calculated solution of a similar system with well-constrained volume and distortion. The obtained mean RMS spot radius is about 0.05 microns, which is shown in Fig. S21. The achieved maximum and average spot sizes of our initial system are in the range of the reported 0.08 and 0.05 microns after optimization (see Table 2 in reference[11]). A further improvement is possible if the user changes to *Free coefficients* mode and clicks *Layout&surfaces* optimization.

A second considered example from their "Larger etendue survey run" has a focal length of 100 mm, an aperture of F/3 and covers a full field of view of 4×4 degree with the stop placed at the first mirror. We have estimated the volume constraint at about 2 liters and the maximum distortion is limited to 1%. This second example (original Fig. 14) is shown in Fig. S22.

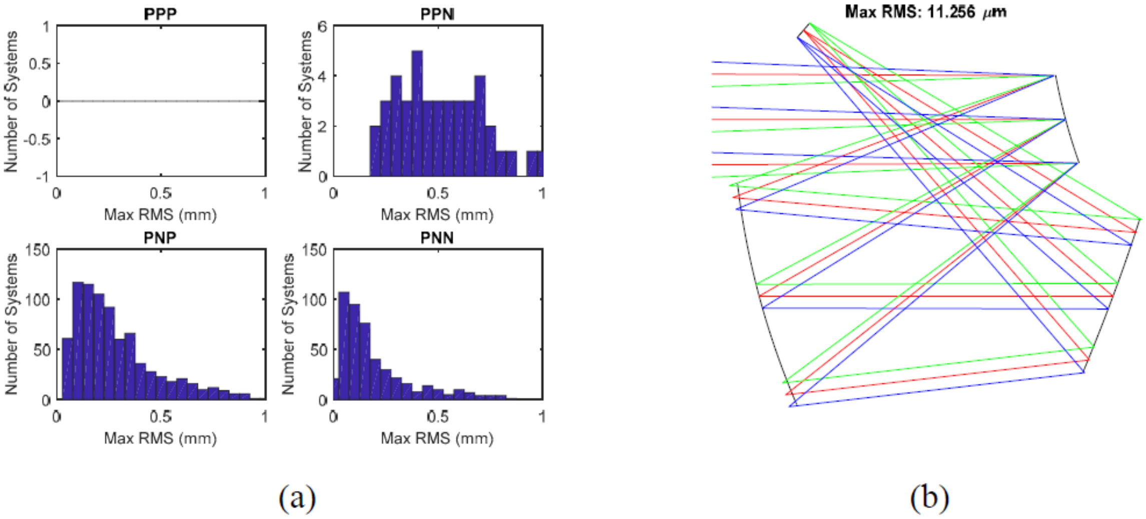

Fig. S22: Original Fig. 14 in reference[11] with caption: "(a) Four performance histograms representing the four distinct tilt angle sign combinations for the survey run with an effective focal length of -100 mm, flat field solution 2, F/3, FFOV 4 degrees by 4 degrees. The systems are binned according to the worst field point RMS spot size. (b) An optical layout of the best performing, analytically designed system from this survey run, along with its max RMS spot size of 11.25 microns."

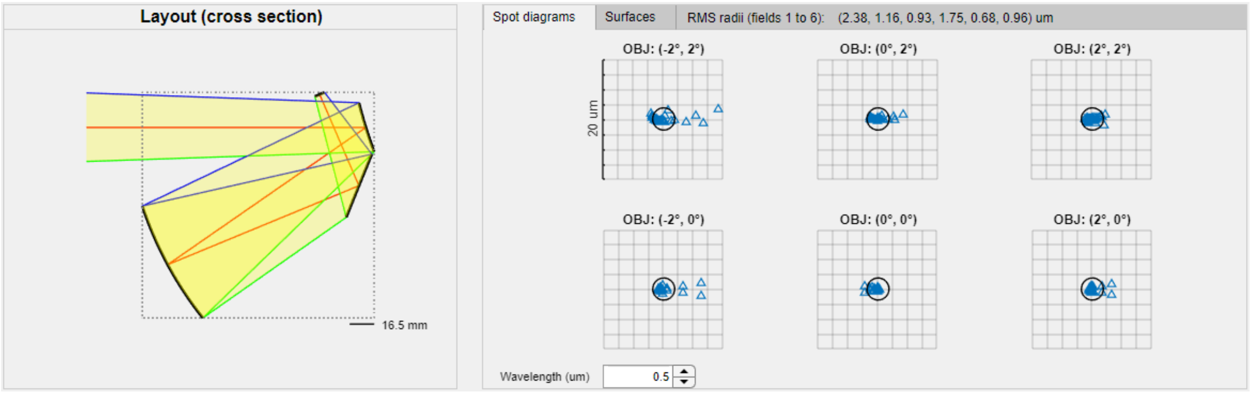

Fig. S23: Reproduced result based on direct calculations for the example (original Fig. 14) discussed in reference[11]

Our redesign of reference example *6b. doi.org/10.1117/12.2314403 (Fig. 14)* of the calculated solution with well-constrained volume and distortion is shown in Fig. S23. The achieved maximum and average spot sizes of about 2.4 and 1.3 microns, respectively, are close to but not yet reaching the reported 1.06 and 0.68 microns of the optimized system (see Table 7 in reference[11]). However, if the user changes to *Free coefficients* mode and clicks *Layout&surfaces* optimization, the optimized result will further improve the resulting performance. It is worth mentioning for the "Larger etendue survey run", that a zig-zag geometry like the one shown in Fig. S21 would allow even better results.

Supplementary References

1. Cai, D. & Gross, H. Obscuration elimination in three-dimensional nonsymmetrical optical systems. *Journal of Physics: Photonics* **1**, 044002 (2019).
2. Bauer, A., Schiesser, E. M. & Rolland, J. P. Starting geometry creation and design method for freeform optics. *Nature Communications* **9**, 1756 (2018).
3. Menke, C. Application of particle swarm optimization to the automatic design of optical systems. *Proceedings of SPIE 10690, Optical Design and Engineering VII*, (SPIE, 2018, 106901A).
4. Yang, T., Jin, G. F. & Zhu, J. Automated design of freeform imaging systems. *Light: Science & Applications* **6**, e17081 (2017).
5. Fuerschbach, K., Rolland, J. P. & Thompson, K. P. A new family of optical systems employing φ-polynomial surfaces. *Optics Express* **19**, 21919-21928 (2011).
6. Muslimov, E. *et al.* Combining freeform optics and curved detectors for wide field imaging: a polynomial approach over squared aperture. *Optics Express* **25**, 14598-14610 (2017).
7. Shafer, D. Three Mirror Freeform design study, https://www.slideshare.net/operacrazy/three-mirror-freeform-design-study (accessed 01/18/2021).
8. Zhong, Y. & Gross, H. Initial system design method for non-rotationally symmetric systems based on Gaussian brackets and Nodal aberration theory. *Optics Express* **25**, 10016-10030 (2017).
9. Zhu, H., Cui, Q., Piao, M. & Zhao, C. Design of a dual-band MWIR/LWIR circular unobscured three-mirror optical system with Zernike polynomial surfaces. *Proceedings of SPIE 9272, Optical Design and Testing VI*, (SPIE, 2014, 92720W).
10. Xu, C., Lai, X., Cheng, D., Wang, Y. & Wu, K. Automatic optical path configuration variation in off-axis mirror system design. *Optics Express* **27**, 15251-15261 (2019).
11. Papa, J. C., Howard, J. M. & Rolland, J. P. Three-mirror freeform imagers. *Proceedings of SPIE 10690, Optical Design and Engineering VII*. (SPIE, 2018, 106901D).
